# The impact of post-processing on spinal cord diffusion tensor imaging

**DOI:** 10.1016/j.neuroimage.2012.12.058

**Published:** 2013-04-15

**Authors:** Siawoosh Mohammadi, Patrick Freund, Thorsten Feiweier, Armin Curt, Nikolaus Weiskopf

**Affiliations:** a Wellcome Trust Centre for Neuroimaging, UCL Institute of Neurology, University College London, UK; b Department of Brain Repair & Rehabilitation, UCL Institute of Neurology, UCL, London, UK; c Spinal Cord Injury Center Balgrist, University Hospital Zurich, University of Zurich, Zurich, Switzerland; d Siemens AG, Healthcare Sector, Allee am Roethelheimpark 2, 91052 Erlangen, Germany

**Keywords:** DTI, Fractional anisotropy, Spinal cord, Eddy current and motion correction, Robust fitting

## Abstract

Diffusion tensor imaging (DTI) provides information about the microstructure in the brain and spinal cord. While new neuroimaging techniques have significantly advanced the accuracy and sensitivity of DTI of the brain, the quality of spinal cord DTI data has improved less. This is in part due to the small size of the spinal cord (ca. 1 cm diameter) and more severe instrumental (e.g. eddy current) and physiological (e.g. cardiac pulsation) artefacts present in spinal cord DTI. So far, the improvements in image quality and resolution have resulted from cardiac gating and new acquisition approaches (e.g. reduced field-of-view techniques). The use of retrospective correction methods is not well established for spinal cord DTI. The aim of this paper is to develop an improved post-processing pipeline tailored for DTI data of the spinal cord with increased quality. For this purpose, we compared two eddy current and motion correction approaches using three-dimensional affine (3D-affine) and slice-wise registrations. We also introduced a new robust-tensor-fitting method that controls for whole-volume outliers. Although in general 3D-affine registration improves data quality, occasionally it can lead to misregistrations and biassed tensor estimates. The proposed robust tensor fitting reduced misregistration-related bias and yielded more reliable tensor estimates. Overall, the combination of slice-wise motion correction, eddy current correction, and robust tensor fitting yielded the best results. It increased the contrast-to-noise ratio (CNR) in FA maps by about 30% and reduced intra-subject variation in fractional anisotropy (FA) maps by 18%. The higher quality of FA maps allows for a better distinction between grey and white matter without increasing scan time and is compatible with any multi-directional DTI acquisition scheme.

## Graphical abstract

**Figure:**
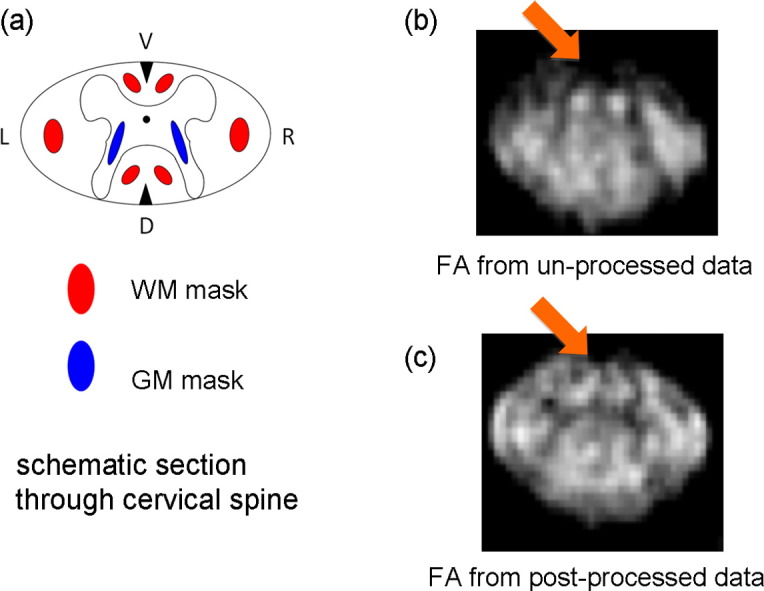

**Highlights:**

► Goal: Reliable definition of grey and white matter in spinal cord DTI. ► Un-processed fractional anisotropy (FA) map is blurry and biassed. ► Processed FA is crisp, unbiased, and contrast-to-noise ratio is improved. ► Retrospective processing of data improves spinal cord DTI.

## Introduction

In the past years, more sophisticated imaging techniques such as functional (Eippert et al., 2009; Lotze et al., 2006; Sprenger et al., 2012; Wietek et al., 2008) and diffusion magnetic resonance imaging (MRI) (Agosta et al., 2007; Budde et al., 2007; Ciccarelli et al., 2007; Mulcahey et al., 2012) have become available for imaging the spinal cord. Diffusion MRI allows for non-invasive tracking of water diffusion (Le Bihan et al., 1986; Turner et al., 1990) and can be used to map brain anatomy (Bach et al., 2011; Basser et al., 1994; Draganski et al., 2011; Mohammadi et al., 2012b; Mueller et al., 2011; Pierpaoli and Basser, 1996). In clinical research diffusion tensor imaging (DTI), a particular implementation of diffusion MRI, has become a wide-spread and successful imaging method (Duning et al., 2009; Keller et al., 2011; Meinzer et al., 2010; Warnecke et al., 2010). For example, the scalar DTI-index denoted as fractional anisotropy (FA) has been reported to be sensitive to white matter integrity in health and disease in the brain (Deppe et al., 2007; Freund et al., 2012b; Pierpaoli et al., 2001) and spinal cord (Agosta et al., 2007; Budde et al., 2007; Ciccarelli et al., 2007; Freund et al., 2012c; Mulcahey et al., 2012).

The spinal cord is a small structure (ca. 1 cm in total diameter) and specific localization of injuries in the spinal cord requires a robust distinction between grey matter (GM) and white matter (WM) (Freund et al., 2012a). Up to now, most diagnostic studies in the spinal cord were limited by the quality and resolution of the DTI reconstruction (e.g. equal to or more than 1 mm^2^ in-plane resolution (Agosta et al., 2007; Budde et al., 2007; Ciccarelli et al., 2007; Freund et al., 2011; Mulcahey et al., 2012; Roser et al., 2010)). Due to the cylindrical symmetry of the spinal cord, usually thick slices (about 5 mm) with maximal in-plane resolution are acquired leading to particularly long EPI readout times (Finsterbusch, 2009b, 2012; Rossi et al., 2008; Wilm et al., 2007, 2009) and making the signal susceptible to physiological and instrumental artefacts. Physiological artefacts caused by bulk motion of the cord and cerebrospinal fluid (CSF) pulsation can result in slice-to-slice displacement, deformation, and signal-loss due to a shift of the echo centre in k-space (Chung et al., 2010; Mohammadi et al., 2012a; Skare and Andersson, 2001). Instrumental artefacts caused by eddy currents (Haselgrove and Moore, 1996; Jezzard et al., 1998; Mohammadi et al., 2010), gradient inhomogeneities (Bammer et al., 2003; Mohammadi et al., 2012d; Nagy et al., 2007), vibration artefacts (Gallichan et al., 2010; Mohammadi et al., 2012c), and RF transmit field inhomogeneities (Lutti et al., 2010, 2012) can lead to image distortions (Mohammadi et al., 2010), affect the diffusion weighting (Mohammadi et al., 2012d), and perturb the signal intensity (Gallichan et al., 2010; Lutti et al., 2010, 2012; Mohammadi et al., 2012c). Up to now, the improvements in image quality and resolution were based on cardiac gating (Rossi et al., 2008; Wheeler-Kingshott et al., 2002a,b) and new acquisition technology, such as reduced field-of-view techniques (Finsterbusch, 2009b, 2012; Rossi et al., 2008; Wheeler-Kingshott et al., 2002a,b; Wilm et al., 2007, 2009), stronger diffusion weighting gradients (Wilm et al., 2009), increased number of averages (Rossi et al., 2008), and time-efficient monopolar diffusion-weighting schemes (Finsterbusch, 2009a; Morelli et al., 2010).

Surprisingly, the use of post-processing correction methods was rarely reported in spinal cord DTI (Barakat et al., 2012; Cohen-Adad et al., 2011; Freund et al., 2012c; Lundell et al., 2013; Wilm et al., 2009). However, using post-processing correction methods could potentially reduce remaining artefacts and even compensate for some of the drawbacks of the reported new acquisition approaches. For example, the methods that are related to improved diffusion weighting (stronger diffusion gradients or monopolar diffusion schemes) usually increase instrumental artefacts such as eddy currents (Haselgrove and Moore, 1996; Jezzard et al., 1998) and could benefit from retrospective eddy current correction (see, e.g., Wilm et al., 2009). Physiological artefacts in DTI affect data quality and can be reduced retrospectively using robust tensor fitting (Mangin et al., 2002; Walker et al., 2011; Zwiers, 2010) and linear modelling of artefacts (Mohammadi et al., 2012a). Increasing the number of averages might lead to more subject motion artefacts, which can be corrected using three-dimensional (3D) affine (e.g. Cohen-Adad et al., 2011; Mohammadi et al., 2010; Muñoz Maniega et al., 2007) or slice-wise (e.g. Mohammadi et al., 2010; Speck et al., 2006) registration methods.

The aim of this paper is to provide an improved processing pipeline for robust DTI in the spinal cord, which is compatible with previously suggested acquisition methods. To this end, we determine the effect of pre-processing (none, 3D-affine, and slice-wise eddy current and motion correction) and tensor estimation (ordinary least squares vs. robust tensor fitting) methods on the image quality and contrast-to-noise ratio (CNR) between GM and WM.

## Methods

### Subjects

Nine healthy adult volunteers (1 female, 8 males, age: 35 ± 8) participated in the study approved by the local ethics committee after giving written informed consent.

### Data acquisition

**Fig. 1:**
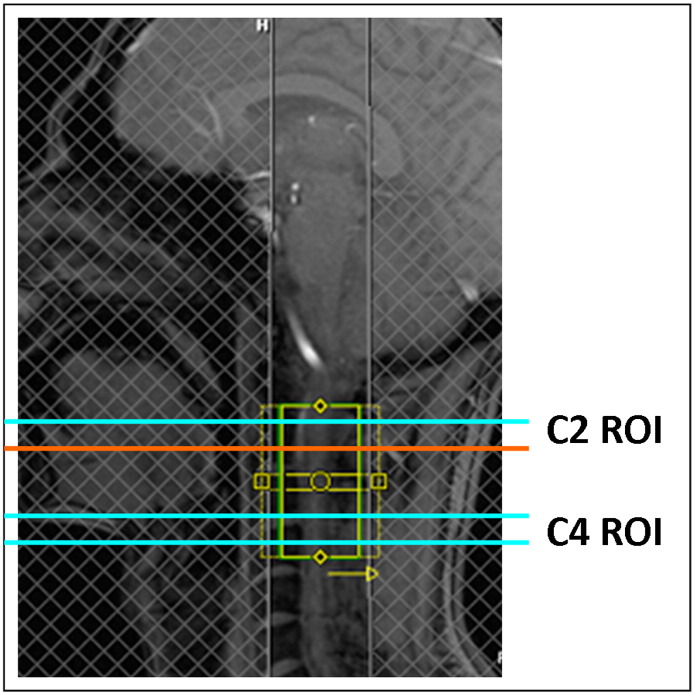
Positioning of the field of view (small central solid green/yellow) covering cervical segments C2 and C4 (sagittal view). Reduced field of view was achieved by minimizing the phase-encoding steps in the anterior-posterior direction and avoiding consequential fold-over by two spatial saturation pulses (shaded regions). The slice positions of the grey and white matter region of interest (ROI) in the upper part of C2 (C2 ROI, cyan and orange horizontal lines) and in the lower part of C4 (C4 ROI, two cyan horizontal lines) are depicted. The grey and white matter ROIs at the position of the orange line are shown in Fig. 2.

Experiments were performed on a MAGNETOM Trio, a Tim System, 3T scanner (Siemens Healthcare, Erlangen, Germany) operated with an RF body transmit coil and a 12-channel (12-ch) receive-only head, 4-ch neck and 24-ch spine coil. Only the 4 neck channels and the 6 posterior head channels were used, since they provided full coverage of the scanned area. DTI data were acquired with a cardiac-gated monopolar diffusion sequence (Morelli et al., 2010) using the following parameters: 30 diffusion-weighted (DW) images (b = 500 s/mm^2^), 5T2-weighted images without diffusion weighting (b = 0 images), 5 mm slice thickness, with 10% inter-slice gap, 10 slices perpendicularly oriented to the spine, 5/8 Partial-Fourier Imaging in phase-encoding direction, phase oversampling 50%, and a cardiac trigger delay of 200 ms. Two slightly different in-plane resolutions, field-of-view (FoV), and echo times (TEs) were used in this study: 176 × 40/176 × 60 acquisition matrix, 123 × 28/128 × 43 mm^2^ FoV, 0.7 × 0.7/0.73 × 0.73 mm^2^ in-plane, echo time of TE = 73/75 ms, slice repetition time of TR = 290/350 ms. The gated data were acquired in blocks of two slices per cardiac cycle. The minimal time between successive triggers was 1800 ms. The reduced FoV was achieved using two saturation pulses (Heidemann et al., 2009) (see Fig. 1). Subjects S1–S6 and S8 were measured with the first set of parameters, and subjects S7 and S9 with the second set of parameters. The difference between the two protocols was small and we did not observe any difference in the resulting image quality. Each DTI dataset was acquired four times, resulting in 140 images for each subject. Altogether, this resulted in a total acquisition time of about 5.8 min (as estimated by the sequence simulator), but could be longer depending on the participant's heart rate. Subsequently, the abbreviations x, y, and z are used for the directions right-left (frequency encoding), anterior-posterior (phase encoding), and head-feed (slice selection), respectively.

### Pre-processing and tensor estimation

First, the in-plane field-of-view was chopped to 28 × 28 mm^2^ for each DTI dataset to exclude non-spine tissue. Next, the images were interpolated to a higher in-plane resolution of 0.35 × 0.35 mm^2^. Finally, the data were corrected for motion and eddy current artefacts using three different registration methods: (a) none, (b) 3D-affine, and (c) combination of rigid-body and slice-wise motion correction (details are summarised in Table 1). The 3D-affine registration corrects for rigid-body subject motion and linear eddy current effects (see (Mohammadi et al., 2010)). Before applying the slice-wise registration, a 3D-affine registration was performed to reduce 3D translation in x- and y-direction as well as scaling effects in y-direction. We restricted the slice-wise registration to correct only for in-plane x- and y-translation as well as for in-plane scaling in y-direction, because we observed most variation in those directions. We did not correct for in-plane rotation and shearing effects, which were less pronounced and more difficult to estimate robustly.

After pre-processing, the FA was estimated using two different tensor-fitting methods: (a) ordinary least squares (Koay et al., 2006) and (b) a new robust-fitting method based on (Mohammadi et al., 2012a; Zwiers, 2010). We extended the robust-fitting method of Zwiers to account also for whole-volume outliers (e.g. due to 3D-affine misregistrations) by introducing an extra Gaussian weighting term that scales with the average of the residuals over the whole volume. The pre-processing and tensor fitting methods are summarised in Table 2. All analysis steps were performed using SPM8 (http://www.fil.ion.ucl.ac.uk/spm (Friston et al., 2006)), the “Artefact Correction in Diffusion MRI (ACID)” SPM toolbox (http://www.fil.ion.ucl.ac.uk/spm/ext), and in-house software written in MATLAB (version 7.11.0; Mathworks, Natick, MA, USA).

### Analysis: comparing different tensor processing techniques

The performance of the pre-processing and tensor estimation methods (see Tables 1 and 2) was assessed by using visual inspection of DW and FA images and quantitative analyses. The quantitative analyses consisted of a modified jackknife approach, and a region-of-interest (ROI) analysis of GM and WM mean FA values, and the CNR between the GM and WM FA ROIs.

### Visual inspection of DW and FA maps using different tensor processing methods

Maps of the estimated DW image in z-direction (along the spinal cord) and FA were calculated for each pre-processing and tensor estimation method. To obtain the estimated z-direction-DW image, the diffusion tensor was fitted and for each DW direction the forward model was applied, i.e. the DW images were calculated based on the estimated diffusion tensor. The DW signal, *S*_25_, acquired with the diffusion gradient, **G**_25_, that had the greatest absolute value in z-direction (**G**_25_ = (0.01, 0.19, − 0.98)) was chosen for visual inspection, because it showed the best contrast between GM and WM due to the cords' special anatomy.

Finally, the root-mean-square (rms) of the tensor-fit errors and the outliers were visualised for one example DTI dataset. To compare the rms-tensor-fit error from the ordinary least square and robust tensor fitting we adjusted these by the degrees of freedom (Mohammadi et al., 2012a), since they varied between the two methods. To quantify the outliers, the robust-tensor-fitting weights within a manually defined FA-based mask were calculated.

### A Jackknife-based assessment of the variance of the FA map for each post-processing method

To calculate a Jackknife-based variance measure of the FA maps for each post-processing method *m* (definition of *m* can be found in Table 2), four additional FA maps (FA_(*m*)_^(*j*)^, *j* = 1,…,4) were estimated on the basis of a subset of only three out of four DTI datasets, where the *j*-th dataset was left out. In total, for each post-processing method *m* five FA maps were calculated: the so-called reference FA map (FA_(*m*)_^*R*^) based on all four DTI datasets and four FA maps based on a subset of only three DTI dataset (FA_(*m*)_^(*j*)^, *j =* 1,…,4). To assess the performance of the post-processing method *m* the variance map was calculated with respect to the reference FA map:

(1) $$\sigma^{2}\left({\mathrm{FA}}_{\left(m\right)}\left(r\right)\right)=\frac{1}{4}{\sum_{j=1}^{4}\left({\mathrm{FA}}_{\left(m\right)}^{R}\left(r\right)-{\mathrm{FA}}_{\left(m\right)}^{\left(j\right)}\left(r\right)\right)}^{2}\text{.}$$

Next, the spatial average of each variance map, *σ*^2^(FA_(*m*)_(**r**)), within the spinal cord was calculated and its square root was taken. This yielded a single variance estimate *σ*FA_(*m*)_ for each post-processing method *m* and subject. To facilitate comparison between the post-processing methods, the relative change of *σ*FA_(*m*)_ with respect to the method (vi) was calculated:

(2) $$\delta {\mathrm{FA}}_{\left(m\right)}=100\times \frac{\sigma {\mathrm{FA}}_{\left(\mathrm{vi}\right)}-\sigma {\mathrm{FA}}_{\left(m\right)}}{\sigma {\mathrm{FA}}_{\left(\mathrm{vi}\right)}}$$

with *m* = (i),…,(v). Note that we chose the method (vi) as the reference method, but in principle any of the methods could have been chosen.

Finally, the *δ*FA_(*m*)_ for each subject was used to calculate group averages (based on the median) and inter-individual variations (based on the standard error of the mean).

Note that the jackknife-based assessment of the post-processing methods is unbiased and does not favour any of the post-processing methods, since it does not require manual definition of ROIs or prior information.

### Group comparison of GM and WM FA-values and FA-based CNR using different tensor processing methods

**Fig. 2:**
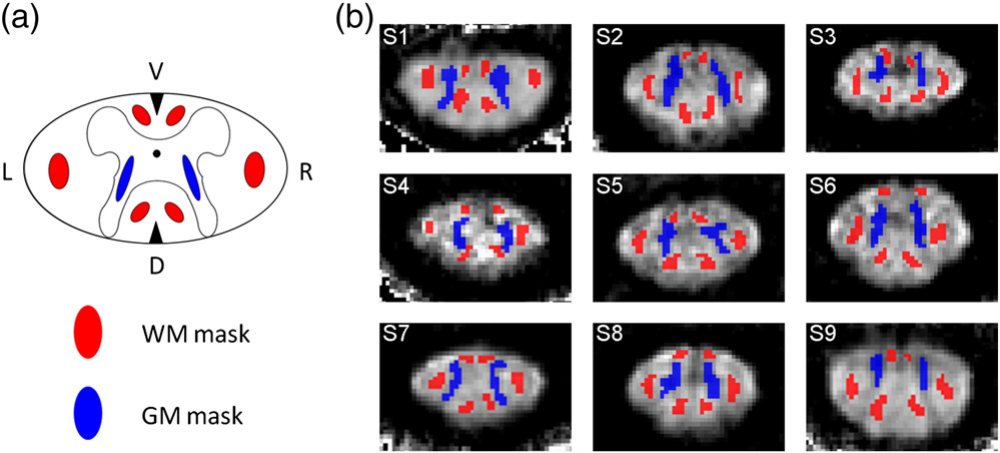
The grey matter (GM) and white matter (WM) region of interest (ROI) depicted in the schematic drawing of the cross-section of the spinal cord (a, GM and WM ROIs were highlighted in blue and red, respectively). The WM ROI covers parts of the left (L) and right (R) lateral funiculus as well as dorsal (D) and anterior (V) columns. The GM ROI covers parts of the butterfly-shaped GM structure. One example slice (slice position is shown in Fig. 1) of the individual WM and GM ROIs is overlaid on the corresponding FA maps for each subject ((b): subject 1–9). The ROIs were manually defined based on the corresponding reference FA image, which was obtained after slice-wise eddy current and motion correction with robust tensor fitting (method (vi), see Table 2).

In these analyses, we used the FA map obtained from method (vi) (slice-wise motion and eddy current correction with robust tensor fitting) as reference. The FA maps obtained from the methods (i)–(v) were registered to the FA map of the reference method (vi) using a rigid-body transformation. First, the mean FA was calculated in the GM $\left({\bar{\mathrm{FA}}}_{\mathrm{GM},\left(m\right)}\right)$ and WM $\left({\bar{\mathrm{FA}}}_{\mathrm{WM},\left(m\right)}\right)$ ROIs using the processing method *m* (see Table 2 for method definition). The GM and WM ROIs were manually defined for each subject based on the FA images obtained from method (vi) (see Fig. 2). Then, the ${\bar{\mathrm{FA}}}_{\mathrm{GM},\left(m\right)}$ and ${\bar{\mathrm{FA}}}_{\mathrm{WM},\left(m\right)}$ values for each subject were used to calculate group averages (based on the median) and inter-individual variations (based on the standard error of the mean).

Next, the CNR was calculated for each subject within the GM and WM ROIs:

(3) $${\mathrm{CNR}}_{\left(m\right)}=\left({\bar{\mathrm{FA}}}_{\mathrm{WM},\left(m\right)}-{\bar{\mathrm{FA}}}_{\mathrm{GM},\left(m\right)}\right)/\left(\sqrt{\sigma_{\mathrm{WM},\left(m\right)}^{2}+\sigma_{\mathrm{GM},\left(m\right)}^{2}}\right)\text{,}$$

with *σ*_WM,(*m*)_^2^, *σ*_GM,(*m*)_^2^ being the variances of the FA across all voxels in the GM and WM ROI, respectively. Moreover, we calculated the relative reduction of the CNR when using the methods *m =* (i)-(v) relative to the method (vi) in percent:

(4) $$\delta {\mathrm{CNR}}_{\left(m\right)}=100\times \left({\mathrm{CNR}}_{\left(\mathrm{vi}\right)}-{\mathrm{CNR}}_{\left(m\right)}\right)/{\mathrm{CNR}}_{\left(\mathrm{vi}\right)}\text{.}$$

Finally, the group median and standard error of the mean (sem) of the CNRs and relative CNRs were calculated for each method *m*.

## Results

### Visual inspection of DW images and FA maps using different tensor processing methods

**Fig. 3:**
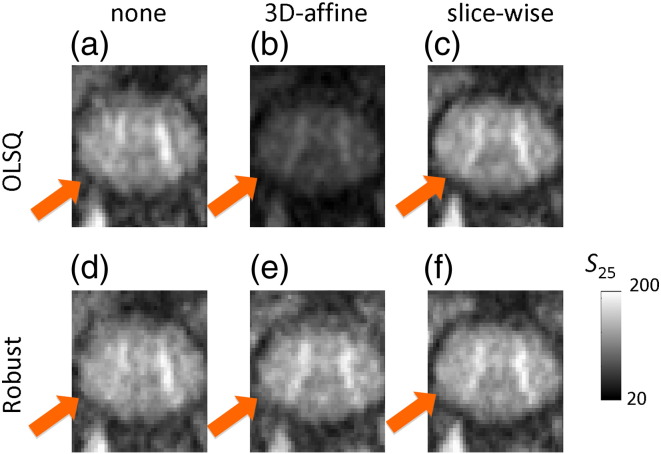
Effect of motion and eddy current correction and tensor fitting methods on the estimated diffusion-weighted (DW) image in z-direction (*S*_25_) using (a)–(f) methods (i)–(vi) as listed in Table 2. The effects are presented for subject 2 in the C2 ROI (slice position is depicted in Fig. 1, see red horizontal line). The left wing of the butterfly-shaped grey matter structure (highlighted) becomes more apparent after motion and eddy current correction (c, e, and f). The 3D-affine motion and eddy current correction (b) spuriously reduced the image intensity of the estimated *S*_25_ map when used in combination with ordinary least square fitting.

**Fig. 4:**
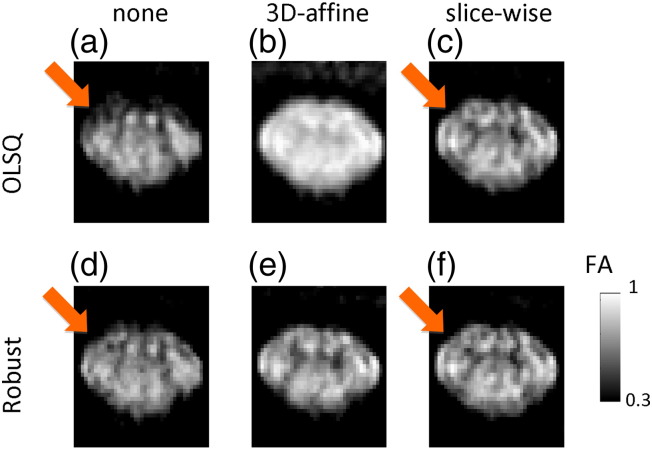
Effect of motion and eddy current correction and tensor fitting methods on the estimated FA maps using (a)–(f) method (i)–(vi). The spatially localised artificial reduction in white matter FA (arrow in (a)) was counteracted when robust tensor fitting (d), motion and eddy current correction (c), or both methods (e and f) were used. The 3D-affine motion and eddy current correction following the use of ordinary least square fitting (b) apparently led to an artificial increase in FA over the whole spinal cord section.

Figs. 3 and 4 exemplify how the different pre-processing and tensor-fitting methods affected the contrast of DW and FA maps in a single subject. Using no pre-processing and ordinary least square tensor fitting resulted in poor contrast between the butterfly-shaped GM and surrounding WM (arrow, Figs. 3a and b) and localised artificial reductions of WM FA (arrow, Fig. 4a), which can lead to a bias in the overall WM FA towards lower values. The slice-wise registration counteracted the localised FA reduction (Figs. 4c and f) and made the butterfly-shaped GM better visible (Figs. 3c and f). Robust tensor fitting also compensated for the artificial FA reduction even if no motion and eddy current correction were employed (arrow, Fig. 3d). The 3D-affine registration, for which some volumes were drastically misregistered (data not shown), led to a signal reduction in the DW images (Fig. 3b) and an artificial increase of the FA over the whole spinal cord section (Fig. 4b). When the proposed robust-fitting method was applied, the misregistration-related volume outliers were down-weighted and the bias in the DW (Fig. 3e) and FA (Fig. 4e) images was removed. Note that the 3D-affine registration did not always lead to a deterioration of the DW and FA image quality, but Figs. 3 and 4 illustrate that it may for some datasets.

### Relating FA-bias to tensor-fit error and outliers

**Fig. 5:**
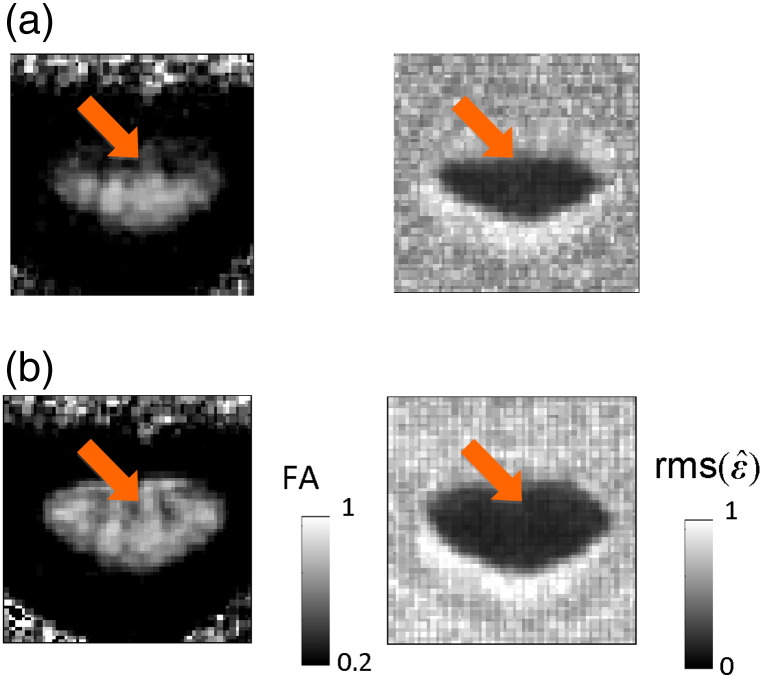
Effect of robust fitting on the adjusted tensor-fit error. FA (left) and the adjusted root-mean-square tensor-fit error ($\mathrm{rms}\left(\hat{\varepsilon }\right)$, right) maps were depicted using (a) method (i), i.e. ordinary least squares, (b) method (iii), i.e. robust tensor fitting. (a) The bias in the FA map (arrow) was associated with a higher tensor-fit error. (b) Robust fitting reduced the bias in the FA map and the associated tensor-fit error. Note that a severely affected slice (z = 2) for the subject S5 was used to visualise the effect of robust fitting on the tensor-fit error.

**Fig. 6:**
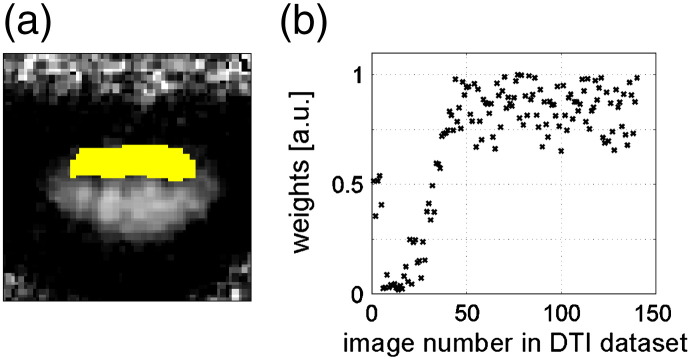
Visualising outliers for the example-slice shown in Fig. 5a: (a) a ROI was manually defined (yellow) within the affected region, (b) robust-tensor-fitting weights within the ROI are depicted as a function of the number of the DTI images arranged as a time-series (i.e. first acquired image = 1, last acquired image = 140). Within the ROI more than 30 (out of 140) DTI images were detected as outliers (i.e. having a weight that is smaller than 0.5). The outliers appeared sequentially in time and thus were probably related to subject motion.

Fig. 5a shows an example-slice, where the WM FA appeared biassed towards lower values. This apparent artefact was associated with a particularly high rms-tensor-fit error (arrow). Both, the bias in WM FA and rms-tensor-fit error, appeared reduced when using robust fitting (Fig. 5b). The rms-tensor-fit error showed that the corrected artefacts were not only present in isolated voxels but also within a continuous region (arrows, Fig. 5). The artefact that was extended over a contiguous region might be due to a misalignment between different DTI images. Fig. 6b shows that from 140 DTI images more than 30 images deviated from the expected value severely (i.e. more than ¼ of the weights were below 0.5). The outliers in the DTI dataset in Fig. 6b were clustered with respect to time, i.e. they appeared one after the other early on in the scan, pointing towards a motion related artefact.

### A jackknife-based assessment of the variance of the FA map for each post-processing method

**Fig. 7:**
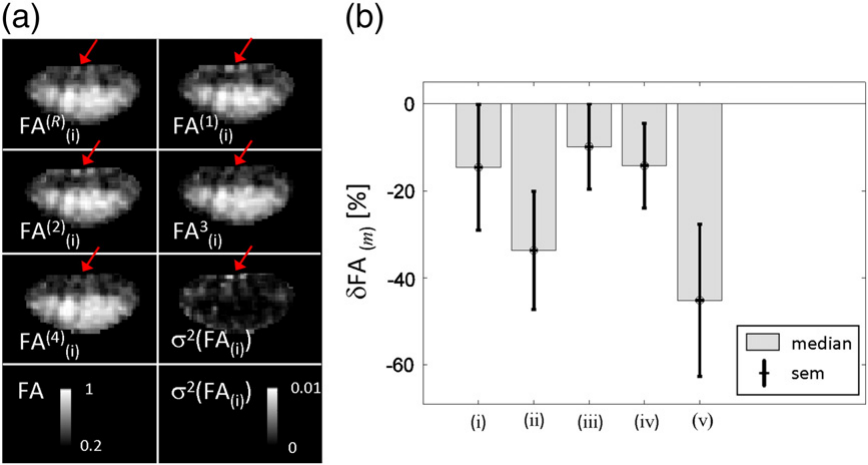
Jackknife-based assessment of variance in FA maps: (a) five FA maps (FA*^R^*_(i)_ and FA^(*j*)^_(i)_, i = 1,…,4) and the corresponding variance maps (σ^2^(FA_(i)_)) are depicted using the post-processing method (i) (i.e. using ordinary least square fitting) for a severely affected slice (z = 2) of the subject S5. The arrows highlight a region with high variance between different FA maps. (b) The group median and standard error of the mean of the spatially averaged variance in the FA map (δFA_(*m*)_) when using post-processing method *m* = (i),…,(v) relative to method (vi). The variance in the FA maps was minimal for the post-processing method (vi). Note that the δFA_(*m*)_ is negative if the variation in the FA maps is higher for the method *m* than for the method (vi).

Fig. 7a depicts an example of five FA maps (FA*^R^*_(i)_, FA^(1)^_(i)_,…, FA^(4)^_(i)_) that were used in the jackknife analysis to assess the variance in FA maps for the post-processing method (i). Regions with a high variance coincided with regions suffering from a high bias in FA (Fig. 7a, arrow). The spatially averaged variation in the FA map was higher (i.e. negative δFA_(*m*)_ in Fig. 7b) when using the post-processing methods (i)–(v) compared to using the method (vi). The variance was increased by about 18% when no post-processing was applied (i.e. method (i) was used). The variance was greatest if the 3D-affine registration was used (i.e. methods (ii) and (v)), which is most likely due to the additional misregistration-related outliers.

### Group comparison of GM and WM FA-values and FA-based CNR using different tensor processing methods

**Fig. 8:**
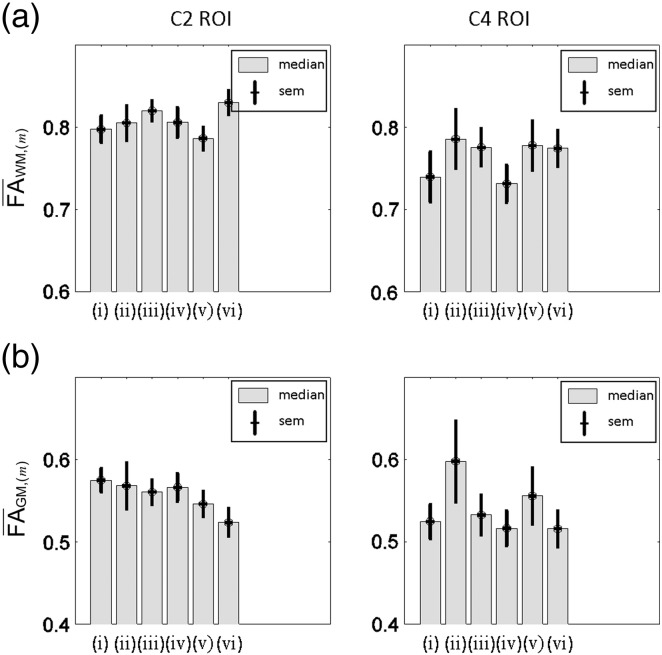
Group level comparison of FA values in white (a) and grey (b) matter ROIs of upper part of C2 (i.e. upper slice in C2 ROI) and lower part of C4 (i.e. lower slice in C4 ROI) when using methods (i)–(vi). For both group comparisons the median and standard error of the mean (sem) across subjects are depicted. Using method (vi) leads to increased WM-FA and decreased GM-FA values.

**Fig. 9:**
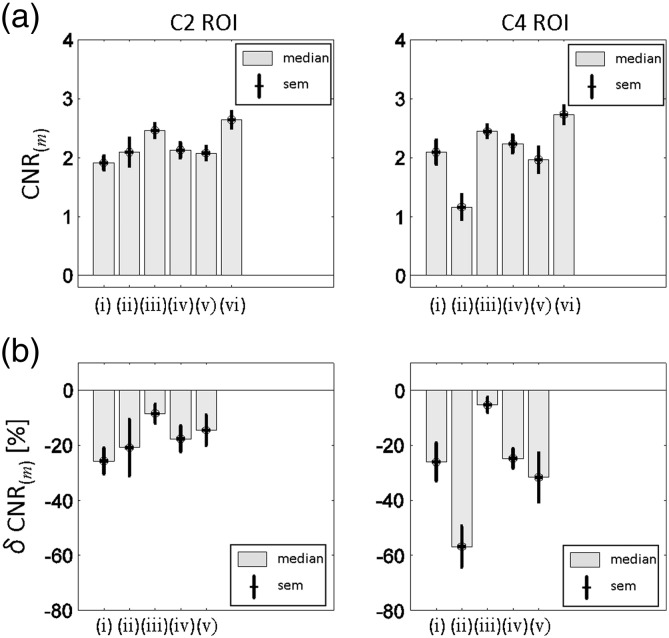
Quantitative assessment of the contrast-to-noise ratio (CNR) between grey and white matter in FA maps of the upper part of C2 (i.e. upper slice in C2 ROI) and lower part of C4 (i.e. lower slice in C4 ROI). (a) Group-level comparison of the CNR for the methods (i)-(vi). (b) Group-level comparison of the relative CNR difference when using method (vi) relative to using methods (i)–(v). For both group comparisons the median and standard error of the mean (sem) across subjects are depicted. The CNR was greater than 2.5 if the recommended post-processing and tensor estimation were used (method (vi)).

Figs. 8 and 9 summarise the effect of different pre-processing and tensor-fitting methods on the mean FA in the GM and WM, and the CNR of FA maps. The WM FA was lowest if no registration (methods (i) and (iv)) was applied (${\bar{\mathrm{FA}}}_{\mathrm{WM},\left(i\right)}$ and ${\bar{\mathrm{FA}}}_{\mathrm{WM},\left(\mathrm{iv}\right)}$ below 0.75, Fig. 8a). The WM FA was highest (${\bar{\mathrm{FA}}}_{\mathrm{WM},\left(\mathrm{vi}\right)}$ above 0.8, Fig. 8a) if slice-wise registration and robust fitting were employed (method (vi)). The GM FA was minimal when slice-wise registration and robust fitting (method (vi)) were applied (${\bar{\mathrm{FA}}}_{\mathrm{GM},\left(\mathrm{vi}\right)}$ about 0.5) and greatest when 3D-affine registration and ordinary least square estimation (method (ii)) were applied (${\bar{\mathrm{FA}}}_{\mathrm{GM},\left(\mathrm{ii}\right)}$ about 0.6, Fig. 8b). The CNR was worst (CNR ≈ 1.5) if the 3D-affine registration and ordinary least square estimation were used (method (ii)) and best (CNR ≈ 3, Fig. 9a) for the slice-wise registration (method (iii) and (vi)). The variation of the CNR values across the group was smallest (sem ≈ 0.1, Fig. 9a) when slice-wise registration was used (methods (iii)). It was maximal (sem up to 0.2) when no registration (method (i)) or 3D-affine registration and the ordinary least squares estimation (methods (ii)) were used. Relative to method (vi) the CNR was reduced by about 30% when no pre-processing and the ordinary-least-squares tensor estimation (i.e. method (i)) was used (Fig. 9b). The CNR was reduced by up to 50% if the 3D-affine registration and ordinary least squares were used (methods (ii), Fig. 9b). Using robust fitting without any pre-processing resulted in a CNR reduction of about 25% (Fig. 9b, (iv)). The smallest CNR reduction (less than 10%) was achieved when the slice-wise registration and ordinary-least-squares tensor estimation (method (iii)) were applied (Fig. 9b).

## Discussion

We tested whether and to what extent different post-processing methods affected the data quality of spinal cord DTI. We found that post-processing can efficiently reduce the noise and increase the CNR in FA maps. However, inappropriate post-processing methods (e.g. 3D-affine registration for spinal cord DTI) can occasionally fail and even introduce additional bias into the diffusion tensor estimates. We introduced a new robust fitting method specifically designed to down-weight misregistration-related outliers. The combination of slice-wise motion correction, eddy current correction, and robust fitting yields the maximal CNR and minimal variation in FA maps. As a result of this improved post-processing, GM and WM can be better distinguished in FA maps and the power of group studies is increased.

The ability to clearly distinguish between GM and WM within the spinal cord holds promise to improve our understanding of pathologies that affect both substructures (e.g. multiple sclerosis (Ciccarelli et al., 2007) or spinal cord injury (Dietz and Curt, 2006; Enck et al., 2006; Lotze et al., 1999, 2006; Wietek et al., 2008)). The results suggest that both types of proposed processing methods (i.e. eddy current and motion correction and robust tensor fitting) reduce potential bias in FA maps (Figs. 4–6). This might be one reason for the increased WM FA values and decreased GM FA values across the group (Fig. 8) when using the proposed method, i.e., slice-wise registration and robust fitting. The latter finding is in accordance with previous studies showing that noise and instrumental artefacts increase the FA in GM (e.g. Mohammadi et al., 2012c,d; Skare et al., 2000). Moreover, the DW signal from the butterfly-shaped GM structure in the spinal cord became more visible against the background noise when a slice-wise registration method was applied (Fig. 3). The robust tensor fitting improved the quality of the DW signal only if global bias was introduced as a result of improper processing (i.e. 3D-affine misregistrations). However, robust fitting did not improve the DW signal locally in the GM. This might be related to the fact that the performance of the robust fitting method depends on the validity of the diffusion tensor model, which imperfectly describes the DW signal in GM structures (Alexander et al., 2006; Miller et al., 2012; Wedeen et al., 2005).

Very few publications have investigated the GM and WM properties of the spinal cord at 3T separately (Finsterbusch, 2012; Maier and Mamata, 2005; Wilm et al., 2007, 2009) and reported DTI indices (e.g. FA) (Wilm et al., 2009). Wilm et al. (2009) achieved CNRs of 1.7 to 2 using an outer-volume suppression method to increase the in-plane resolution. (Note that the CNR was not reported in Wilm et al. (2009), we calculated it using their reported mean and standard deviation of the FA in the cervical spine's WM and GM). In our study, the CNRs were about two if no correction was applied (and even smaller, CNR < 1.5, if 3D-affine registration and ordinary least squares were used). The CNRs, however, clearly exceeded two (CNR > 2.5, i.e. about 30% improvement) if slice-wise motion and eddy current correction together with robust tensor fitting were employed. This finding demonstrates that unprecedented quality of spinal cord DTI can be achieved when appropriate DTI-processing methods are used. Note that the comparison of CNRs obtained from different scanners and DTI sequences should be treated with caution, because deviations in the details of the diffusion weighting (gradient duration δ, diffusion time Δ, gradient amplitude), variation in instrumental artefacts (see e.g. Mohammadi et al., 2012c) as well as differences in the b values, resolution, RF pulses etc. could have an influence on the FA values and CNR estimates.

Spinal cord imaging is susceptible to instrumental artefacts due to the high demands on the scanner hardware (i.e. very high in-plane resolution and unilateral (dorsal) radio-frequency receive coil coverage) and to physiological artefacts due to a small structure of interest with bone-CSF-GM-WM transitions that is subject to CSF pulsation, swallowing, cardiac or respiratory motion. This makes the robust estimation and reproducibility of DTI indices particularly difficult and complicates clinical and high-end research application of spinal cord DTI. In this study, we found that the inter-subject variance of the CNR was reduced by a factor of up to two and the intra-subject variance in FA maps was reduced by about 18% if appropriate tensor processing was used. This finding suggests that the proposed retrospective artefact correction methods facilitate reliable high-resolution spinal cord DTI and thus might be beneficial for clinical research.

To achieve improved eddy current and motion correction, different pre-processing steps were necessary: (a) chopping the field-of-view to eliminate non-spine tissue, (b) interpolation to higher in-plane spatial resolution to increase effective resolution of the tensor estimates, and (c) slice-wise correction of a minimum of affine spatial transformation parameters which explained most of the distortion. Unlike the slice-wise registration, the 3D-affine eddy current and motion correction that worked successfully when applied on brain-DTI data (e.g. (Mohammadi et al., 2010; Muñoz Maniega et al., 2007)) could lead to misregistrations and introduce a bias in the FA values on spinal cord DTI data. One reason for a difference in the performance of 3D-affine vs. slice-wise registration is image distortions due to physiology-related motion in the x-y plane that can vary along the slice-select direction (z-axis; Yiannakas et al., 2012). Yiannakas et al. (2012) achieved significant improvement in image quality by reducing the amount of non-rigid-body motion during spinal cord MRI using a cervical collar. In spinal cord DTI, the acquisition time from the first to the last slice is about 30 times longer (10 × TR = 2900 ms) than the acquisition time for one single slice (about 100 ms), leading to differential movement perpendicular to the z-axis direction and non-rigid-body-like image distortions. These non-rigid-body-like distortions can be approximated piecewise by linear transformations along the x- and y-direction (here we used translation and scaling) and thus effectively corrected using a slice-wise registration.

Despite the latter problem, 3D-affine registration is often applied to spinal cord DTI data during post-processing. One approach to address the misregistrations is to remove volume-outliers by manual user intervention. However, this approach might affect the reproducibility and suffers from selection bias. Here we present an alternative approach, which uses the robust-tensor-fitting framework to down-weight volume-outliers and thus to correct for bias in the estimated DW and FA images.

Instead of correcting eddy current image distortions retrospectively, an eddy current compensated diffusion sequence can be used, e.g., the twice-refocusing spin-echo sequence (Reese et al., 2003). We acquired one DTI pilot dataset using the twice-refocusing spin-echo sequence and discarded this option for eddy current compensation in this study, because it led to an increase in echo time by about 22% and thus to a significant reduction of the signal to noise ratio (data not shown).

The optimum between numbers of non-collinear and collinear (i.e. averaged) diffusion directions is controversially discussed (Hasan, 2007; Jones and Basser, 2004; Santarelli et al., 2010). In spinal cord DTI often the minimum of six non-collinear diffusion directions and a maximum amount of averages are acquired (e.g. Finsterbusch, 2012; Rossi et al., 2008; Wilm et al., 2009). Here, we acquired four averages and 30 different diffusion directions. Our approach is motivated by the fact that the robust fitting method works best for more than 30 diffusion directions (Chang et al., 2005).

While motion and eddy current correction methods only reduce geometrical misalignment of the images (Mangin et al., 2002; Mohammadi et al., 2010), the robust tensor fitting approach down-weights outliers in the diffusion signal (Chang et al., 2005; Mohammadi et al., 2012a; Zwiers, 2010) and thus corrects for both, image-intensity and geometrical-misalignment-driven outliers (Figs. 5 and 6). Robust fitting might be of particular interest for DTI data in the spinal cord, where physiological effects can lead to signal-modulations, which bias the diffusion signal, and to local (non-linear) deformations that cannot be addressed by (affine) image registration methods. Furthermore, robust fitting minimizes the impact of error-prone processing step (e.g. 3D-affine registrations for spinal cord DTI). However, robust fitting methods have to be treated with caution, because they can lead to a less stable tensor fit (i.e. an increased condition number in the matrix inversion) and thus to noise enhancement (see, e.g., Mohammadi et al., 2012a; Skare et al., 2000), if too many data points are down-weighted. Furthermore, if the ordinary-least-square estimation of the diffusion tensor, which is used as a baseline, is strongly biassed, the robust tensor fitting might fail. Therefore, we recommend using first slice-wise registration to reduce the misalignment within the DTI dataset and thus to improve the ordinary-least-square tensor estimation, and afterwards robust fitting to down-weight residual misalignment artefacts and outliers in the diffusion signal.

While the CNR analysis was important to show the advantage of the processing for the DTI data (e.g. to improve DTI data quality), one might argue that the ROI definition on the basis of the FA images using method (vi) may result in a somewhat circular CNR analyses (Fig. 9). In particular, it may favour method (vi), which served as the anatomical reference. To show, that the method (vi) most effectively reduced the noise and bias in the data, we employed a jackknife analysis, which does not favour any post-processing method and yielded the same principal results.

We note that the image acquisition parameters for subject 7 and 9 were slightly different from the rest. It could be argued that the different parameters may have led to small differences in data quality, which in theory could bias our findings. However, we also performed all group analyses excluding subject 7 and 9 (data not shown) and observed the same fundamental results (i.e. best results were obtained using slice-wise eddy current and motion correction and robust fitting). Thus, we considered the effect of slightly different acquisition parameters to be negligible.

## Conclusion

Post-processing in spinal cord DTI is possible and should be applied, because it allows for better distinction between grey and white matter within the spinal cord, and reduces the intra- and inter-subject variance. In clinical studies involving spinal cord pathologies and high-end research, where reliable results are crucial and scan time is limited, the use of the proposed robust tensor processing might be of particular benefit.

## Figures and Tables

**Table 1:** The 3D-affine registration corrects for rigid-body subject motion and 3D eddy currents (see Mohammadi et al., 2010). The slice-wise registration was preceded by a rigid-body registration to reduce 3D shifts in the x- and y-direction, and 3D scaling in the y-direction.

Registration method	Translation	Rotation	Scaling	Shearing	Number of parameters
3D-affine	x-, y-, and z- direction	x-, y-, and z- axis	x- and y- direction	x-y plane and z-y plane	9
Rigid-body	x- and y-direction	None	y-direction	None	3
Slice-wise	x- and y-direction	None	y-direction	None	24^a^

a Due to poor data quality and edge effects, the first and last slices were not included in the slice-wise registration method, yielding 8 × 3 parameters.

**Table 2:** Six different combinations of post-processing methods.

Method	Eddy current and motion correction	Tensor estimation
(i)	None	Ordinary least squares
(ii)	3D-affine	Ordinary least squares
(iii)	Slice-wise	Ordinary least squares
(iv)	None	Robust fitting
(v)	3D-brain	Robust fitting
(vi)	Slice-wise	Robust fitting
